# GmSAL1 Hydrolyzes Inositol-1,4,5-Trisphosphate and Regulates Stomatal Closure in Detached Leaves and Ion Compartmentalization in Plant Cells

**DOI:** 10.1371/journal.pone.0078181

**Published:** 2013-10-22

**Authors:** Yee-Shan Ku, Nicolas Siu-Chung Koo, Francisca Wing-Yen Li, Man-Wah Li, Hongmei Wang, Sau-Na Tsai, Feng Sun, Boon Leong Lim, Wing-Hung Ko, Hon-Ming Lam

**Affiliations:** 1 Center for Soybean Research, State Key Laboratory of Agrobiotechnology and School of Life Sciences, The Chinese University of Hong Kong, Shatin, N.T., Hong Kong SAR; 2 School of Biological Sciences, The University of Hong Kong, Hong Kong SAR; 3 School of Biomedical Sciences, The Chinese University of Hong Kong, Shatin, N.T., Hong Kong SAR; RIKEN Center for Sustainable Resource Science, Japan

## Abstract

Inositol polyphosphatases are important regulators since they control the catabolism of phosphoinositol derivatives, which are often signaling molecules for cellular processes. Here we report on the characterization of one of their members in soybean, GmSAL1. In contrast to the substrate specificity of its *Arabidopsis* homologues (AtSAL1 and AtSAL2), GmSAL1 only hydrolyzes inositol-1,4,5-trisphosphate (IP_3_) but not inositol-1,3,4-trisphosphate or inositol-1,4-bisphosphate.The ectopic expression of *GmSAL1* in transgenic *Arabidopsis thaliana* led to a reduction in IP_3_ signals, which was inferred from the reduction in the cytoplasmic signals of the *in vivo* biomarker pleckstrin homology domain–green florescent protein fusion protein and the suppression of abscisic acid-induced stomatal closure. At the cellular level, the ectopic expression of *GmSAL1* in transgenic BY-2 cells enhanced vacuolar Na^+^ compartmentalization and therefore could partially alleviate salinity stress.

## Introduction

Phosphoinositol derivatives play a key role in mediating cellular signals which are often related to abscisic acid (ABA) and calcium signaling pathways [1–3]. Inositol polyphosphatases are therefore potential regulators of cellular processes [1–4]. Early characterizations of inositol polyphosphatases were mainly conducted in animal systems and have successfully identified inositol polyphosphatases with different substrate specificities [4,5]. Besides phytases which act on inositol hexakisphosphate (IP_6_; phytate) [6,7], there are two major classes of inositol polyphosphatases identified in plants: inositol 5-phosphatases and inositol 1-phosphatases [8–10]. 

In *Arabidopsis thaliana*, a total of 15 genes were predicted to encode for inositol 5-phosphatases [11], based on the presence of two consensus domains (Domain I and Domain II) identified by aligning characterized inositol 5-phosphatases from animals, yeast and plants [4]. Despite sequence homology at the two consensus domains, the proteins encoded by these 15 *Arabidopsis* genes show little overall sequence similarity, suggesting a diverse group of inositol 5-phosphatases present in plants [11]. Plant inositol 5-phosphatases also exhibit different substrate specificities. For example, At5PTase1 and At5PTase2 can hydrolyze inositol-1,4,5-trisphosphate (IP_3_) and inositol-1,3,4,5-tetrakisphosphate (I(1,3–5)P_4_) [10,11] while At5PTase3, At5PTase7, and At5PTase11 all act on phosphatidylinositol-4,5-bisphosphate (PI(4,5)P_2_) and phosphatidylinositol-3,5-bisphosphate (PI(3,5)P_2_) [12,13]. At the same time, At5PTase11 can also use phosphatidylinositol-3,4,5-trisphosphate (PI(3–5)P_3_) as a substrate [12].

Gain-of-function experiments showed that the overexpression of *At5PTase1* resulted in a reduction of the stomatal response toward light and ABA treatment, presumably due to a lowered IP_3_ level [8]. The overexpression of *At5PTase2* also showed decreased sensitivity toward ABA inhibitory effects on seed germination [10]. These two pieces of evidence point to the regulatory role of inositol 5-phosphatases in ABA signaling. On the other hand, the overexpression of *At5PTase7* in *A. thaliana* enhanced salt tolerance while the knock-out mutant of *At5PTase7* aggravated salt damage due to a loss in the ability to induce reactive oxygen species that are required to trigger the expression of ABA-responsive genes [13].

The AtSAL1 protein from *Arabidopsis thaliana* was a prototype of inositol 1-phosphatases in plants [14]. AtSAL1 was originally identified as a homologue of the yeast HAL2 protein [9]. *In vitro* enzymatic assays showed that AtSAL1 acts on inositol-1,4-bisphosphate (I(1,4)P_2_) and inositol-1,3,4-trisphosphate (I(1,3,4)P_3_) [9] while IP_3_ is apparently not a preferred substrate [14]. However, a mutation in *AtFRY1* (the same gene as *AtSAL1*) resulted in increased levels of cellular IP_3_. It was proposed that this observation was due to the accumulation of I(1,4)P_2_ and I(1,3,4)P_3_ that inhibited the catabolism of IP_3_ [14].

The *in vivo* functions of AtSAL1 on stress responses are still controversial. The ectopic expression of the *Arabidopsis* gene *AtSAL1* in yeast conferred lithium tolerance, similar to the effects of overexpressing the endogenous *ScHal2* gene in yeast [9]. It was an expected result since *AtSAL1* and *ScHal2* are homologues. However, it was subsequently reported that the overexpression of *AtSAL1* in *A. thaliana* did not elevate NaCl tolerance [15]. In fact, AtSAL1 is a negative regulator of drought tolerance in *A. thaliana*, since a mutation in the *AtSAL1* gene led to enhanced drought tolerance [16].

In this work, we identified the coding sequence of the SAL1 homologue in soybean, GmSAL1. We also characterized its substrate specificity and demonstrated its effects on various stress responses in plant cell through its function as an inositol polyphosphatase.

## Materials and Methods

### Cloning of *GmSAL1*

Soybean (*Glycine max* L. Merr.) plants were grown in a greenhouse. For experiments leading to the cloning of *GmSAL1*, the seeds were first germinated in sand irrigated with water. After the opening of the first trifoliate, the seedlings were irrigated with modified Hoagland’s solution [17]. NaCl treatment was performed using 150 mm NaCl for 3 d. 

Total RNA samples were obtained using a modified phenol extraction protocol [18]. The first-strand cDNA was then obtained from the total RNA by reverse transcription using the Moloney murine leukemia virus-reverse transcriptase (Gibco BRL, Grand Island, NY, USA) according to the manufacturer's manual. Degenerate primers (5'GTNCANGTIGCIIGAYTAYGG3' and 5'GCRTGITCCCAIATYTTYTC3') (N = A/C/G/T; Y = C/T; R = A/G; I = Deoxyinosine) were designed based on the multiple alignments of the following proteins: ScHal2 from *S. cerevisiae* (GenBank accession number: AAR89916); AtSAL1, AtSAL2 and AHL from *A. thaliana* (GenBank accession number: Q42546, NP_201205 and NP_200250 respectively); and RHL from rice (GenBank accession number: Q40639). PCR using the above primer pair successfully amplified a fragment of ~600 bps, under the following conditions: 94 °C 5 min; 50 cycles of 94 °C 1 min, 54 °C 1 min and 72 °C 1 min; followed by 72 °C 5 min; in a 25 µl reaction mixture composed of 5.0 µl of the first-strand cDNA, 5.0 mM MgCl_2_, 0.2 mM dNTPs, 0.8 µM of each primer, 0.5 U *Taq* DNA polymerase (Roche, Indianapolis, IN, USA), and 1× PCR buffer.

The DNA sequence of the full-length coding region of *GmSAL1* was subsequently obtained by 5' and 3' Rapid Amplification of cDNA Ends (RACE) using the SMARTRACE cDNA amplification kit (Clontech Laboratories, K1612, Mountain View, CA, USA), according to the manufacturer's protocol. Gene-specific primers (GSPs)/nested GSPs for 5' and 3' RACE were5'ACCACCTTCAGATTTACCACCGTC3’/5’TGCTTTGACACCGAGTTTTTCTGC3' and 5'GTTGTATTGGGGTGTCTTGGCTTG3’/5’TGTCAAAGCACCACCCAGTCAGAA3'respectively. The *GmSAL1*cDNA clone covering the entire coding region was amplified from the first-strand cDNA samples using the primers 5'CGCCGCTGACACTAATCGTTT3' and 5'CGAGCCGACAACAAAGTTAGC3'. The DNA sequence information of *GmSAL1* was deposited into GenBank (accession number: EF637045).

### DNA sequencing and sequence analysis

DNA sequencing was performed using the ABI PRISM dRhodamine Terminator Cycle Sequencing Ready Reaction kit (PerkinElmer, Waltham, MA, USA) and analyzed by the Genetic Analyzer ABI Prism 3100 system, according to the manufacturer's protocol. Homologue searches were performed with Position-Specific Iterated Basic Local Alignment Search Tool (PSI-BLAST) (http://www.ncbi.nlm.nih.gov/BLAST/). Multiple alignments were performed using the ClustalW program [19] in the BioEdit package (ver. 7.0.5.3).

### Gene expression under stress

Soybean seeds were germinated in vermiculite with water in a greenhouse. After one week, seedlings were transferred to hydroponic cultures with half-strength Hoagland’s solution [20]. Just after the emergence of the first trifoliate, the plants were transferred to half-strength Hoagland’s solution supplemented with 60mM, 125mM, and 185mM NaCl, and 10%, 14%, and 16.5% (w/v) polyethylene glycol (PEG)-6000 for 24h. Treated sample tissues were harvested and frozen in liquid nitrogen for total RNA extraction. A total of 20μg RNA for each sample was used for northern blot analysis. The osmolarity of NaCl and PEG solutions was measured by Advanced^TM^Micro Osmometer (Model 3300; Advanced Instruments, Inc., Norwood, MA, USA).

### Northern blot analysis and real time RT-PCR

Northern blot analysis was performed as previously described [21]. Antisense single-stranded digoxygenin- (Roche, Indianapolis, IN, USA)labeled DNA probes were obtained by PCR according to the manufacturer's manual. The *GmSAL1* or *GFP* cDNA subcloned into the pBluescript II KS (+) vector was used as the template and the primers used in the PCR were 5'AATTAACCCTCACTAAAGGG3' (T3) & 5'GTAATACGACTCACTATAGGGC3' (T7) for the first round and the T3 primer for the second round of amplification.

Real-time PCR was performed according to a previous report [22] using the CFX96 Touch™ Real-Time PCR Detection System (Bio-Rad, Hercules, CA, USA). The following primers were used: 5’-ATTGGGTGTCTTGGCTTGTC-3’ (forward primer for *GmSAL1*), 5’-TGTGTAGAACCACCCAGTGC-3’ (reverse primer for *GmSAL1*), 5’-GGCCTTGTATAATCCCTGATGAATAAG-3’ (forward primer for *AtUBQ10*; house-keeping gene for *A. thaliana* samples) [23], 5’-AAAGAGATAACAGGAACGGAAACATAGT-3’ (reverse primer for *AtUBQ10*) [23], 5’-CCCCTCACCACAGAGTCTGC-3’ (forward primer for *L25*; house-keeping gene for *N. tabacum* samples) [24], 5’-AAGGGTGTTGTTGTCCTCAATCTT-3’ (reverse primer for *L25*) [24].

### Construction of *GmSAL1*transgenic *A. thaliana* lines

Transgenic *A. thaliana* ectopically expressing *GmSAL1* was constructed according to a previous report [22]. The cDNA of *GmSAL1* was cloned into a binary vector [25] downstream of the cauliflower mosaic virus 35S promoter. Six-week-old *A. thaliana* (Col-0) plants were transformed by the vacuum infiltration method[26] using the *Agrobacterium tumefaciens* strain GV3101 (pMP90) transformed with the *GmSAL1* construct. The expression of *GmSAL1*in the transformed *A. thaliana* was verified by real-time PCR (Figure S1 in File S1).

### Construction of *A. thaliana* lines expressing both *PH*_*PLCd*_ *-*GFP and *GmSAL1*

Reciprocal crosses were performed between the *PH*_*PLCd*_ *-GFP* and the *GmSAL1* transgenic lines. Five- to six-week-old plants grown on soil were used. Mature flowers were detached from the pollen donor parent. Sepals, petals, and stamens of the flower buds of the pollen recipient parent were removed with a pair of fine forceps. Pollens of the donor parents were transferred to the stigma of the recipient flower bud. About 2-3 weeks after artificial crossing, seeds were harvested. After a few generations of self-fertilization, double homozygous lines were screened by PCR. For the line *PH*_*PLCd*_ *-GFP/GmSAL1-1*,*GmSAL1* was the pollen donor. For the line *PH*_*PLCd*_ *-GFP/GmSAL1-2*, *PH*_*PLCd*_ *-GFP* was the pollen donor.

### Expression and purification of the GmSAL1 protein in *E. coli*

The coding sequence of *GmSAL1* was amplified by PCR using *Pfx* polymerase (Invitrogen, Carlsbad, CA, USA) with the following primers: 5'CCCCAGATCTATGCCTTACGAGAAGGAATTC3' and 5'CCCGCAATTGTCACAAGGATGAAACTTTC3'. The amplified *GmSAL1*cDNAwas subcloned into pGEX-2T vector (GE Healthcare, Chalfont St Giles, UK) to form a fusion protein with the glutathione S-transferase (GST). The GST-*GmSAL1* construct was then introduced into the *E. coli* strain BL21 (DE3) cells. The expression of the recombinant protein was induced by the addition of 0.1mM IPTG to the *E. coli* culture followed by incubation for 4h, before the cells were washed and resuspended in the lysis buffer (50mM Tris-HCl pH 7.5, 100mM NaCl and 1mM phenylmethylsulfonyl fluoride). The soluble protein fraction was obtained by sonication and subsequent centrifugation. Soluble GmSAL1 protein was affinity-purified by GST-Trap column (Amersham Biosciences, Piscataway, NJ, USA) and then dialyzed overnight in the enzyme assay buffer (25mM Tris-HCl pH 7.5, 1mM MgCl_2_) with 2mM dithiothreitol.

### Enzyme assays and determination of the K_m_ values

Phosphatase assays were performed according to previous reports [9,27,28] with slight modifications. A 100μl reaction mixture containing the recombinant protein and substrate in the assay buffer (25mM Tris-HCl pH7.5 and 1mM MgCl_2_) was incubated at 37°C for 30min and the released inorganic phosphate was quantified at 650nm using a 96-well microtiter plate reader (Tecan Group Ltd., Seestrasse, Männedorf, Switzerland). Protein concentrations were determined by the Bradford method [29]. The K_m_ for IP_3_ hydrolysis was determined by measuring the rate of hydrolysis at the following substrate concentrations: 0.0125mM, 0.025mM, 0.05mM, 0.1mM, and 0.2mM.The K_cat_ of IP_3_ was calculated using the K_m_, protein concentration, and molecular weight of the recombinant protein. All substrates used were from Sigma-Aldrich Co. (St Louis, MO, USA) except I(1,3,4)P_3_ and I(1,4)P_2_ (Echelon Biosciences Inc., Salt Lake City, UT, USA).

### Relative *in vivo* IP_3_ levels in guard cells

The microscopic analysis of relative *in vivo* IP_3_ level in guard cells was according to a previous report [30]. The lower epidermal of rosette leaves of 4-week-old*A. thaliana* grown on soil at 22°C (16h light-8h dark cycle) was peeled off. The epidermal peels were immersed in buffer containing 50μM CaCl_2_ , 5mM KCl, 10mM MES-Tris, (pH 6.15) for 2 h under constant light, before subjected to confocal microscopic analysis. Images were collected using Olympus FV1000(Ex: 488nm; Em: 510–525nm). The fluorescence signals were analyzed using the ImageJ program (ver. 1.371.44p)[31].

### Stomatal aperture assay

The stomatal aperture assay was performed according to a previous report [8]. Leaves of 4-week-old*A. thaliana* grown on soil at 22°C (16h light-8h dark cycle) were detached and incubated in a perfusion solution (50mMKCl, 10mM MES, pH 7.0) without supplements for 2h, followed by incubation in a perfusion solution with supplements for another 2h. In the control experiment, the perfusion solution was supplemented with 0.1% (v/v) MeOH (solvent of ABA). In the other two sets of experiments, perfusion solutionscontaining100μM ABA with or without 5mM CaCl_2_ were used. The concentration of ABA employed was according to a previous report [8]. All incubations were conducted at 22°C under constant light. The differential interference contrast (DIC) images of guard cells were captured using a light microscope (Nikon Eclipse 80i). The stomatal aperture was measured using a digital ruler available in the software SPOT Advance (ver. 4.6, Diagnostic Instruments, Inc.).

### Seed germination assay

The seed germination assay was performed according to a previous report [8].*A. thaliana* seeds were surface-sterilized, placed on half-strength MS agar plate (1% (w/v) sucrose supplemented with one of the following: 0.1% (v/v) MeOH alone, 2.5μM ABA in 0.1% (v/v) MeOH, or 4μM ABA in 0.1% (v/v) MeOH) and kept at 4°C in the dark for 2 d. Seeds were then allowed to germinate at 25°C under continuous light. The germination rate was calculated using 138 to 211 seeds from three independent experiments.

### Establishment of transgenic tobacco BY-2 cell lines

The same recombinant construct used to transform *A. thaliana* was transformed into the tobacco BY-2 cells [32] using *Agrobacterium* (strain LBA4404) by a co-cultivation method [33]. After selecting the transformants on antibiotic-containing medium, PCR screening using gene-specific primers was performed to verify the successful integration of the transgene into the genome and real-time PCR was performed to verify the expression of the transgene in the transformed cell lines. Cells were grown in a liquid MS medium [32] at room temperature in the dark with mild agitation.

### Microscopic analysis of Na^+^ compartmentalization

For Na^+^ compartmentalization studies, BY-2 cells were harvested 4 d after subculture and used for all microscopic analyses. After the cells had been treated with 150mM NaCl in MS medium, they were incubated with shaking at room temperature for 1h. SodiumGreen^TM^ indicator (S6901; Invitrogen, Carlsbad, CA, USA was used to visualize the intracellular contents of Na^+^ [17], and the confocal images were captured using Olympus FV1000 (Ex: 488nm; Em: 510–525nm). The fluorescence signals were analyzed using the ImageJ program (ver. 1.44p)[31]. The total fluorescence intensity in the pixels was divided by the total area to obtain the average pixel fluorescence intensity. Background fluorescence intensity was measured in the same field and was subtracted. DIC images of cells were obtained by excitation with a red diode. Two replicates of each experiment were performed. 

For real-time image capturing, 3-day-old cells were pre-incubated with 10μM Sodium Green^TM^ indicator for 30 min prior to the 200mMNaCl treatment. Images of cells were captured at 10-sec intervals after NaCl treatment for a total period of 50min, using the Bio-Rad Radiance 2100 system (Ex: 514nm, filter set HQ545/40). The first reading was taken ~20s after NaCl treatment. Cell sizes were measured by the ImageJ program (ver. 1.37) and the % change was reported. Na^+^ content was measured as the intensity of the Sodium Green^TM^ signal per unit area using the same program. The images were collated and converted to an MPEG video and attached as a supplementary file.

### Microscopic analysis of cell viability

For cell viability assays, 4-day-old BY-2 cell suspension cultures were remained untreated or treated with one of the following: 150mM NaCl in MS medium, 150mMNaCl with 1μM IP_3_ in MS medium, or 13.3% (w/v) PEG-6000 in MS medium (near-isotonic to 150mM NaCl), for 24 h with shaking in an orbital shaker. After treatment, cells were stained with 0.4μg/μl Trypan blue (Sigma Aldrich Co., St Louis, MO, USA). The images of stained cells (around 150 cells in each experiment) were captured using the CCD camera attached to the light microscope (Nikon Eclipse 80i). A total of 91-247 cells were counted from 4-12 fields. Two replicates of each experiment were performed. 

### NaCl and PEG stress treatments on *A. thaliana*

 Stress treatments on *A. thaliana* were performed as described in previous reports [34,35] with slight modifications. Ten-day-old *A. thaliana* seedlings grown on MS agar at 22°C (16h light-8h dark cycle) were transferred to MS agar without supplement (CK) or MS agar supplemented with 100mM NaCl, 11.1% (w/v) PEG-6000, 150mM NaCl, or 13.5% (w/v) PEG-6000 (100mM NaCl MS broth is near-isotonic to 11.1% (w/v) PEG-6000 MS broth; 150mMNaCl MS broth is near-isotonic to 13.5% (w/v) PEG-6000 MS broth).The *A. thaliana* seedlings were harvested10 days after treatment. The experiments were replicated.

### Determination of total chlorophyll in *A. thaliana*

The determination of total chlorophyll in *A. thaliana* was performed as described previously [34]. Leaf tissue of less than 0.02 g was immersed in 0.8 ml N, N-dimethylformamide (DMF) followed by incubation at 4°C overnight[36]. The absorbance at 603, 647 and 664 nm was recorded. The amount of total chlorophyll was calculated using a formula published previously[37].

### Statistical analysis

Statistical analysis was performed using the Statistical Package for Social Sciences (version 16.0; SPSS Inc., Chicago, IL, USA).

## Results

### The expression of *GmSAL1* was induced by NaCl but not near-isotonic PEG

**Figure 1:**
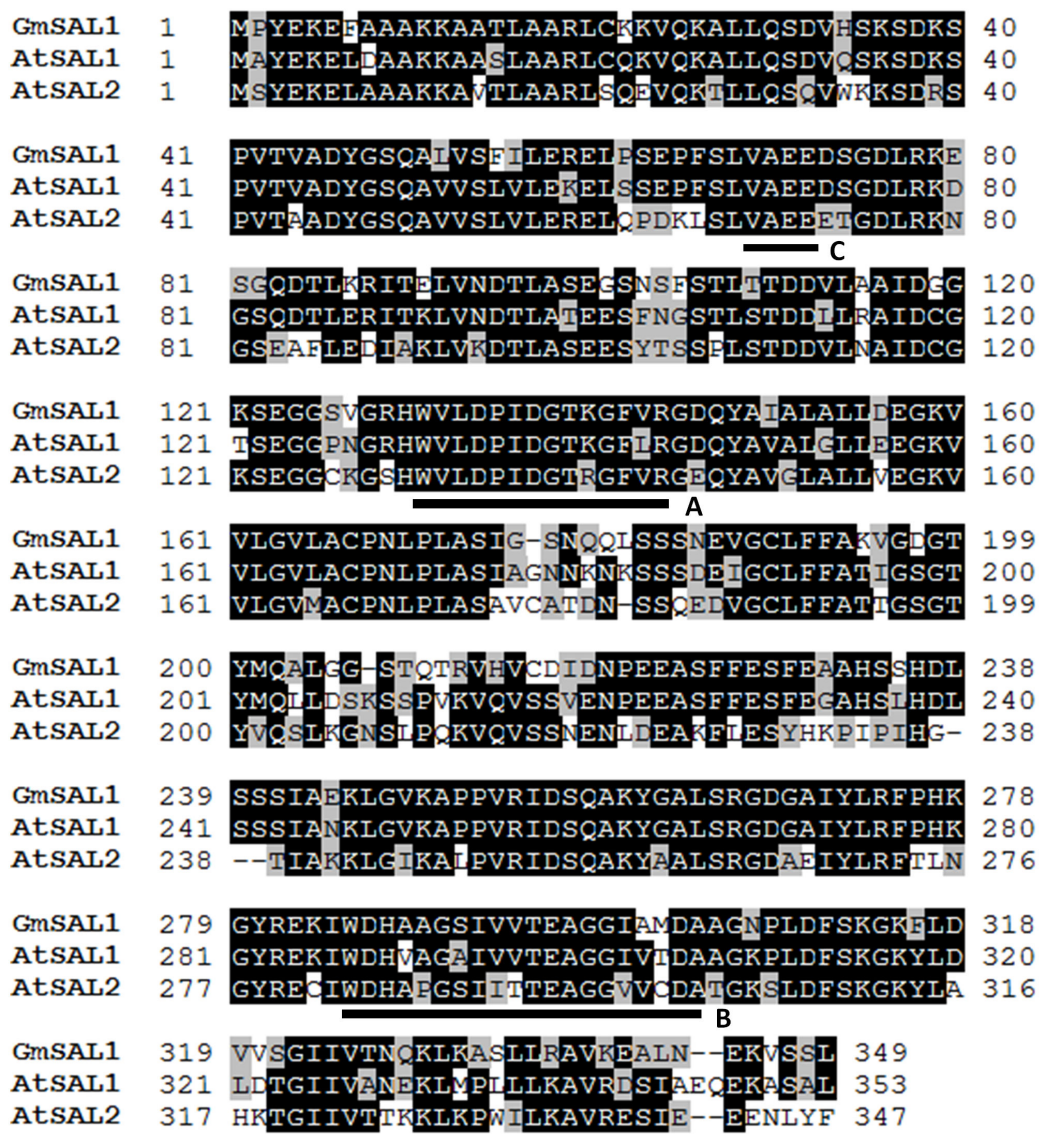
Aligning GmSAL1 with its closest homologues in *A. thaliana*. Multiple alignments were performed for GmSAL1, AtSAL1, and AtSAL2 (Genbank numbers EF637045, Q42546, and NP_201205, respectively), using the ClustalW program [19] in the BioEdit package (ver. 7.0.5.3). Identical amino acid residues were shaded black and similar amino acid residues were shaded grey. The conserved motifs involved in substrate- and metal-binding and nucleophilic water activation were marked as A, B and C [38].

We obtained the full-length coding region of*GmSAL1* (the soybean homologue of*AtSAL1*) by PCR using degenerate primers followed by RACE.Basic Local Alignment Search Tool (BLAST) analysis showed that the overall amino acid sequence identity of GmSAL1 (GenBank accession No.: EF637045) to the closest homologues in *A. thaliana*, AtSAL1 (GenBank accession No.: Q42546) and AtSAL2 (GenBank accession No.:NP_201205), is 77% and 63% respectively. Multiple alignments were performed on GmSAL1, AtSAL1 and AtSAL2 (Figure 1). The consensus sequences [38] for inositol- and phosphate-binding are all conserved in GmSAL1.

**Figure 2:**
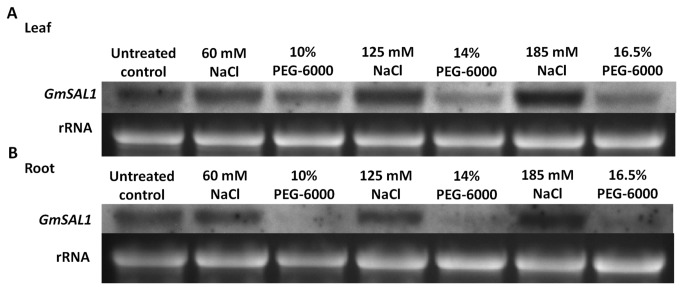
Northern blot analyses of the expression of *GmSAL1* in soybean under NaCl and isotonic PEG-6000 treatments. A series of NaCl solutions with different concentrations were prepared, together with the corresponding near-isotonic PEG solutions: 60mM NaCl versus 10% (w/v) PEG-6000; 125mM NaCl versus 14% (w/v) PEG-6000; 185mM NaCl versus 16.5% (w/v) PEG-6000 (the osmolarities of treatment solutions are given in Table S1 in File S1). Two-week-old soybean seedlings grown hydroponically were placed in fresh half-strength modified Hoagland’s solution without (untreated) or with various NaCl or PEG supplements. Leaf and root tissues were harvested 24 h after treatment. 20μg total RNA from each sample was used for northern blot analysis. The experiment was performed twice and similar results were obtained.

Since the yeast homologue, ScHal2, is known to be a salt-stress determinant [39],we tested the expression of *GmSAL1* when the plants were subjected to different concentrations of NaCl (Figure 2). It was found that the levels of *GmSAL1* transcripts in both leaves and roots were induced by treating the plants with NaCl (Figure 2). Since NaCl treatment consists of two stress components: ionic stress and osmotic stress, we therefore used near-isotonic PEG treatments to control for the osmotic stress. The difference in *GmSAL1* expression levels between each isotonic pair of NaCl and PEG treatments showed that GmSAL1 could only be induced by ionic stress (due to NaCl) but not osmotic stress (due to PEG) (Figure 2). PEG treatment actually led to a repression of *GmSAL1*gene expression (Figure 2).

### GmSAL1 hydrolyzed IP_3_

The amino acid sequence alignment suggested that GmSAL1 may possess inositol polyphosphatase activities(Figure 1). We expressed and purified the GmSAL1 protein from *Escherichia coli* in order to determine its substrate specificity *in vitro*. Surprisingly, we found that GmSAL1 used IP_3_ readily as the substrate but had no effect on I(1,3,4)P_3_, I(1,4)P_2_, inositol 1-monophosphate (I(1)P) or IP_6_. The K_m_ and K_cat_ values were also determined (Table 1). The GmSAL1 substrate specificity is therefore completely different from that of AtSAL1, an inositol 1-phosphatase and the closest homologue of GmSAL1 in *A. thaliana*.

**Table 1:** Substrate specificity (K_m_) and K_cat_ values of GmSAL1^**a**^.

**Substrates**	**Specific activity** (μmol min^-1^mg^-1^)	**K_m_** (μM)	**K_cat_** (min^-1^)
IP_3_	1.1 ± 0.2	10.8± 0.1	68 ±13
I(1,3,4)P_3_	0	-	-
I(1,4)P_2_	0	-	-
I(1)P	0	-	-
IP_6_	0	-	-

a Enzymatic activities were measured as described [9,27,28] in 25 mM Tris-HCl pH7.5, 1 mM MgCl_2_ at 37°C for 30 min. All substrates were in a final concentration of 0.5 mM except IP_3_ (0.2 mM). The results were from three independent experiments, each performed in triplicates. Details for the determination of K_m_ and K_cat_ values were described in Materials and Methods. Numerical data represent the mean value of independent experiments±standard deviation.

 To study the physiological significance of the enzymatic activities of GmSAL1 *in vivo*, we employed the biosensor construct Pleckstrin homology domain–green florescent protein (PH_PLCd_-GFP). PH_PLCd_ is a protein domain which binds to PI(4,5)P_2_ (on the plasma membrane) and IP_3_ (in the cytosol) [30]. The PH_PLCd_-GFP construct was successfully employed to indicate the cytoplasmic IP_3_ levels [30].

 We first generated homozygous lines of transgenic *A. thaliana* expressing *GmSAL1* and confirmed the expression of the transgene (Figure S1 in File S1). Two independent *GmSAL1* transgenic lines were crossed to an *A. thaliana* line expressing the *PH*_*PLCd*_ *-GFP* construct. Homozygous transgenic lines containing both the *GmSAL1* and *PH*_*PLCd*_ *-GFP* constructs were obtained and the expression of *GmSAL1* was validated (Figure S1B in File S1).

 Since the expression of *PH*_*PLCd*_ *-GFP* was reduced in the *GmSAL1*/*PH*_*PLCd*_ *-GFP* double transformants (Figure S1D in File S1), instead of comparing the total GFP signals, we examined the percentage of signals localized in the cytoplasm of guard cells.

**Figure 3:**
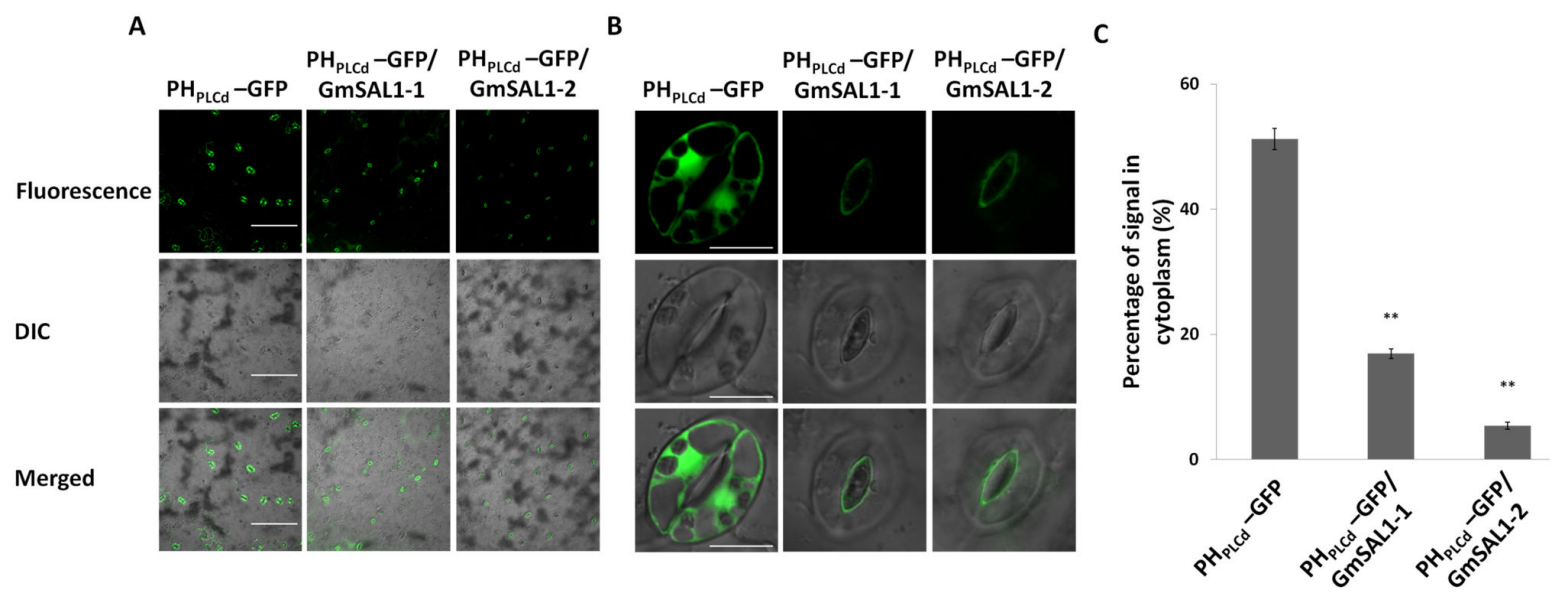
*In* *vivo* changes in IP_3_ signals due to the ectopic expression of *GmGAL1* in *A. thaliana*. The biosensor PH_PLCd_ was employed to study the changes in IP_3_ signals as described in Materials and Methods. Representative fields of the lower epidermis of the original *PH*_*PLCd*_ *-GFP* line and two independent double-transformed *A. thaliana* lines (*PH*_*PLCd*_ *-GFP/GmSAL1-1* and *PH*_*PLCd*_ *-GFP/GmSAL1-2*) under a confocal microscope were shown. The GFP signal is represented by a pseudo-green color. **A**: A low magnification showing a wider view, scale bar= 100μm. **B**: A close-up view of the guard cells, scale bar = 10μm. **C**:Statistical analysis of the percentage GFP signal in cytoplasm of the guard cells. Results were calculated from 30 cells from 6 fields. Error bar: standard error. ** indicates a significant difference (p<0.01) between the original *PH*_*PLCd*_ *-GFP* construct and the double transformants (*PH*_*PLCd*_ *-GFP/GmSAL1*-1or*PH*_*PLCd*_ *-GFP/GmSAL1-2*), using one-way analysis of variance (ANOVA) followed by the posthoc Tukey’s test. The experiment was performed twice and similar results were obtained.

Compared to the original *PH*_*PLCd*_ *-GFP* transgenic line, the proportion of signals in the cytoplasm was much lower in the *GmSAL1*/*PH*_*PLCd*_ *-GFP* double transformants (Figure 3). Together with the *in vitro* enzymatic data, this *in vivo* evidence supports the function of GmSAL1 to down-regulate the level of cytosolic IP_3_. 

### Ectopic expression of GmSAL1 negated the effects of ABA on stomatal closure and seed germination

**Figure 4:**
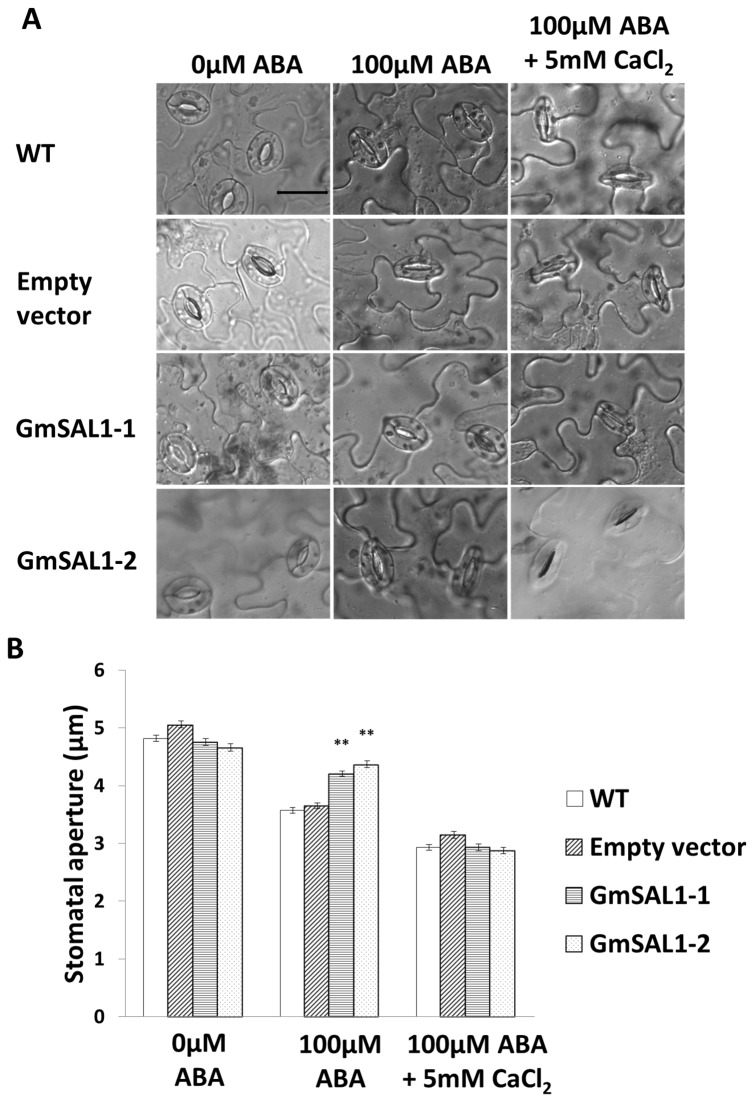
Ectopic expression of *GmSAL1* in *A. thaliana* negated the effects of ABA on stomatal aperture. Leaves from 4-week-old *A. thaliana* grown on soil were used in this experiment. **A**:Representative DIC images of the guard cells of untransformed wild type (WT), empty-vector transgenic control (Empty vector), and two *GmSAL1* transgenic lines (GmSAL1-1 and GmSAL1-2), treated with 0μM ABA, 100μM ABA, or 100μM ABA + 5mM CaCl_2,_ were captured using a light microscope. Scale bar =20μm. **B**: Mean stomatal aperture was measured using a digital ruler. N>168 from repeated experiments. Error bar: standard error. ** indicates a significant difference (p<0.01) between GmSAL1-1 or GmSAL1-2 and WT, based on ANOVA followed by the posthoc Tukey’s test.

 IP_3_ plays a key role in mediating the ABA signaling in guard cells to control the stomatal aperture [8]. To test whether the ectopic expression of *GmSAL1* will also affect IP_3_-mediated stomatal closure, a stomatal aperture assay was conducted. Detached *A. thaliana* leaves were treated in a buffer containing 0.1% (v/v) MeOH, with or without 100μM ABA. Under ABA treatment, the stomatal apertures in the wild type leaves (WT) and the empty vector-transformed control were much reduced compared to no ABA treatment (Figure 4). On the other hand, the stomatal apertures in the leaves of the *GmSAL1* lines were significantly larger than those in the controls under the same ABA treatment (Figure 4).

 The model of ABA-induced stomatal closure postulates that ABA increases the cytosolic IP_3_ level in guard cells, which in turn leads to an increase in cytosolic calcium [Ca^2+^]_cyt_, resulting in the differential activation and inactivation of K^+^ channels on the plasma membrane and the tonoplast [1]. The net result is the efflux of K^+^ (and subsequently water) out of the cytosol and the vacuole, which leads to the loss of turgidity in guard cells and, consequently, stomatal closure [1,3,40]. To investigate whether the effect of GmSAL1 on stomatal aperture is Ca^2+^-dependent, 5mM Ca^2+^ was also included in the medium in addition to 100μM ABA. External Ca^2+^ leads to the elevation in [Ca^2+^]_cyt_ and stomatal closure [41]. Our results indicated that the suppressing effect of GmSAL1 on stomatal closure under 100μM ABA was mitigated by the addition of external Ca^2+^ (Figure 4).In the medium containing 100μM ABA and 5mM Ca^2+^, the stomatal aperture of wild type, the empty-vector line, and the *GmSAL1*transgenic lines show no significant differences (Figure 4).The effect of GmSAL1 on the stomatal opening may hence be a result of its hydrolytic activities toward cellular IP_3_. 

**Figure 5:**
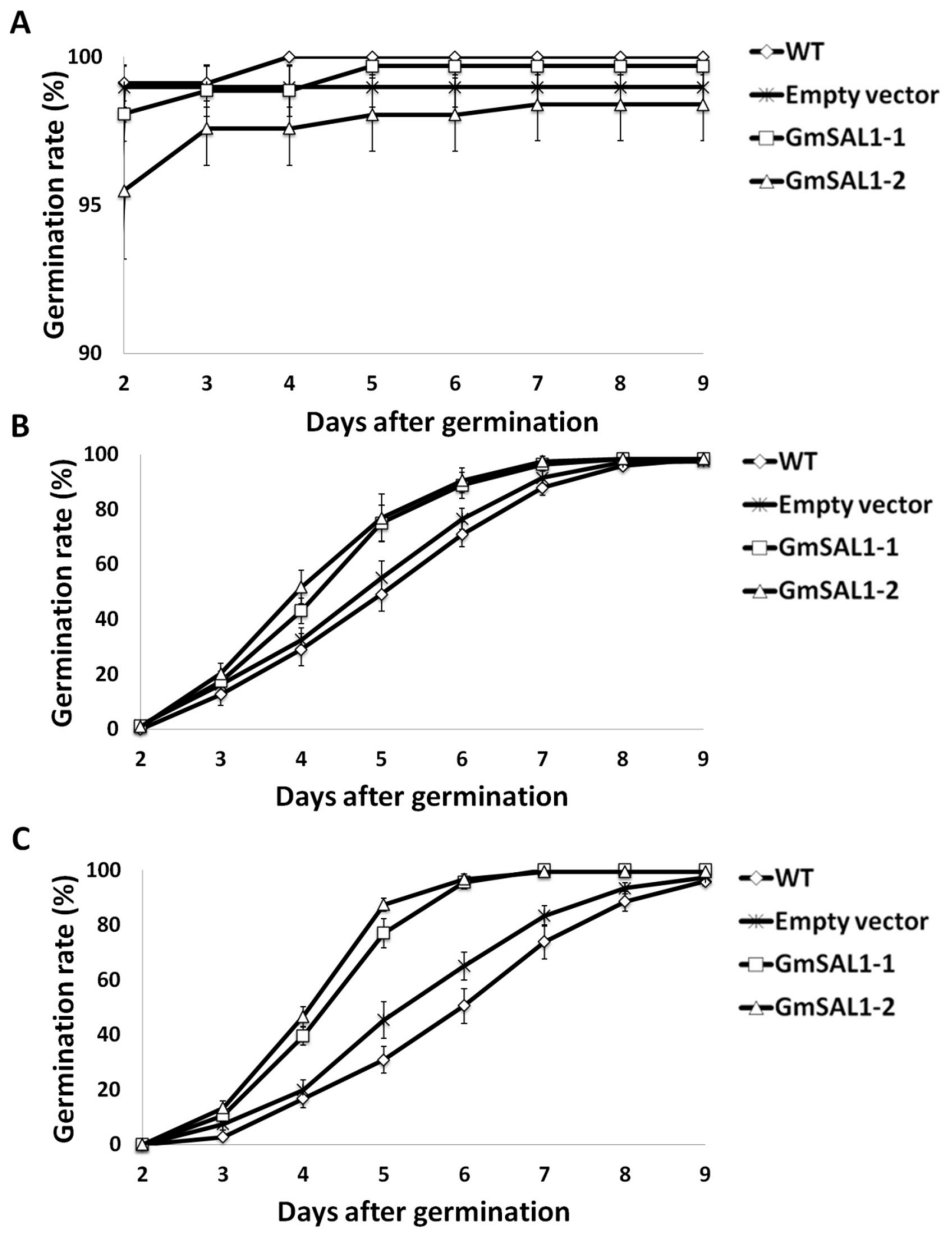
Ectopic expression of *GmSAL1* in *A. thaliana* negated the effects of ABA on seed germination. The germination rates of *A. thaliana* seeds of untransformed wild type (WT), empty-vector transgenic control (Empty vector), and *GmSAL1*-expressing lines, treated with 0μM (**A**), 2.5μM (**B**), or 4μM (**C**) ABA on half-strength MS agar plates, were determined. Results were calculated from 138-211 seeds from three independent experiments. Error bar: standard error. The differences of germination rates among WT, empty vector and *GmSAL1* overexpressing lines were subjected to one-way analysis of variance (ANOVA) followed by the posthoc Tukey’s test. Under 2.5μM ABA treatment, p<0.01 from day 4 to day 7. Under 4μM ABA treatment, p<0.01 from day 4 to day 8.

 Besides controlling the stomatal aperture, another important function of ABA in plants is the inhibition of seed germination. Germination rate is a common strategy to study ABA sensitivity [8,10]. The effects of ABA on the seed germination rate of the wild type *A. thaliana*, empty-vector transgenic control and the*GmSAL1*transgenic lines were compared. Under 2.5μM ABA and 4μM ABA treatments, the germination rate of *GmSAL1*transgenic lines was significantly higher than the controls (Figure 5) with the effects being more pronounced under 2.5μM ABA than 4μM ABA treatment, indicating that GmSAL1 can reduce the sensitivity of plants toward ABA.

### Ectopic expression of *GmSAL1* enhanced vacuolar Na^+^ compartmentalization in BY-2 cells under salinity stress

**Figure 6:**
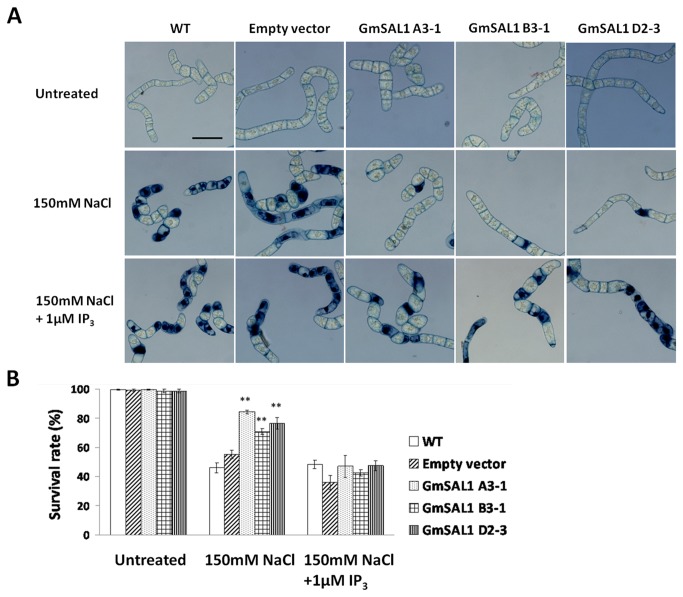
Ectopic expression of *GmSAL1* in BY-2 cells enhanced their survival rates under NaCl stress. Four-day-old BY-2 cells grown in MS medium were used, including cells of untransformed wild type (WT), empty-vector transgenic control (Empty vector), and three independent *GmSAL1* transgenic lines (A3-1, B3-1, D2-3). The survival rate was determined by Trypan blue staining. The cells were untreated, treated with 150mM NaCl or 150mM NaCl+ 1μM IP_3_ in MS medium for 24 h with shaking. They were then washed with fresh MS medium and stained with 0.4μg/μl Trypan blue for 15min before microscopic analysis. **A**: Typical photos showing the rate of survival. The nuclei of dead cells were stained blue. Scale bar= 100μm.B: Statistical analysis. A total of 94-247cells were counted from 4-6 fields. Error bar: standard error. ** indicates a significant difference (p<0.01)between transgenic cells and WT, based on ANOVA followed by the posthoc Tukey’s test.

Next, we examined the effects of GmSAL1 on plant cells in general. A previous study of AtSAL1 showed that its ectopic expression in yeast cells could increase salinity tolerance [9]. Since the expression of *GmSAL1* is salt-inducible (Figure 2), the effect of GmSAL1 on salt tolerance in plant cells was investigated. The survival rates of *GmSAL1* transgenic BY-2 cells under NaCl stress (Figure 6) and PEG-induced osmotic stress (Figure S2 in File S1) were studied. The expression of *GmSAL1* in the transgenic cells was validated (Figure S1 in File S1). Trypan blue was used to stain the dead cells. NaCl (salinity stress) significantly increased the number of dead cells. The expression of *GmSAL1* could alleviate NaC1-induced salinity stress (Figure 6) but not PEG-induced osmotic stress (Figure S2 in File S1). The percentage of survival in the *GmSAL1* transgenic BY-2 cell lines under NaCl stress was significantly higher than in other lines (Figure 6), whereas the supplementation of 1μM IP_3_ could negate the protective effects of GmSAL1 (Figure 6). 

**Figure 7:**
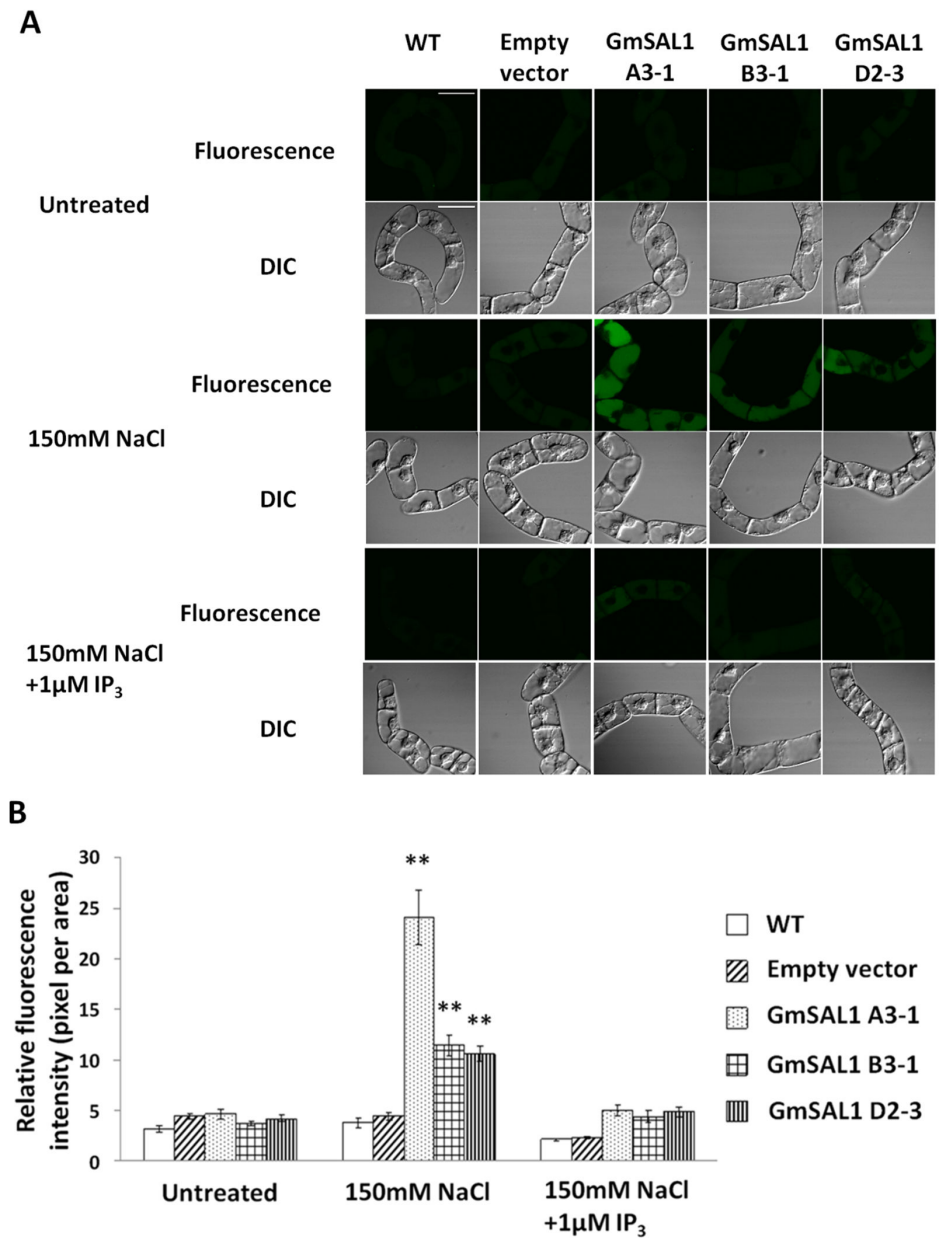
The ectopic expression of *GmSAL1* in BY-2 cells increased the vacuolar compartmentalization of Na^+^ under NaCl stress. Four-day-old BY-2 cells grown in MS medium were used, including cells of the untransformed wild type (WT), empty-vector transgenic control (Empty vector), and three independent *GmSAL1* transgenic lines (A3-1, B3-1, D2-3). Vacuolar Na^+^ compartmentalization was visualized with the use of Sodium Green^TM^. Cells pre-washed with MS medium were transferred to fresh MS medium containing no supplements (untreated), MS medium supplemented with 150mM NaCl or 150mM NaCl+ 1μM IP_3_ for 1h with shaking. They were then washed with fresh MS medium and stained with 5μM Sodium Green^TM^, followed by confocal microscopic analysis. **A**: Typical photos showing the relative levels of vacuolar Na^+^ using the fluorescent signal of Sodium Green^TM^ (represented by the pseudo-green color). Scale bar= 50μm.B: Statistical analysis. The relative fluorescence intensity of 17-34 cells (from 4 fields) was determined for each data point. Error bar: standard error. ** indicates a significant difference (p<0.01) between the transgenic lines and WT, based on ANOVA followed by the posthoc Tukey’s test.

 We also traced the cellular compartmentalization of Na^+^ using the fluorescence dye Sodium Green^TM^. Our results indicated that under NaCl treatment, *GmSAL1* transgenic BY-2 cells exhibited enhanced vacuolar compartmentalization of Na^+^, as reflected by the higher fluorescence intensity in the vacuole when compared to the wild type BY-2 cells and empty-vector transgenic control (Figure 7). Similar to the results of the cell survival test, the effects of expressing GmSAL1 was much reduced by the supplementation of 1μM IP_3_(Figure 7).

**Figure 8:**
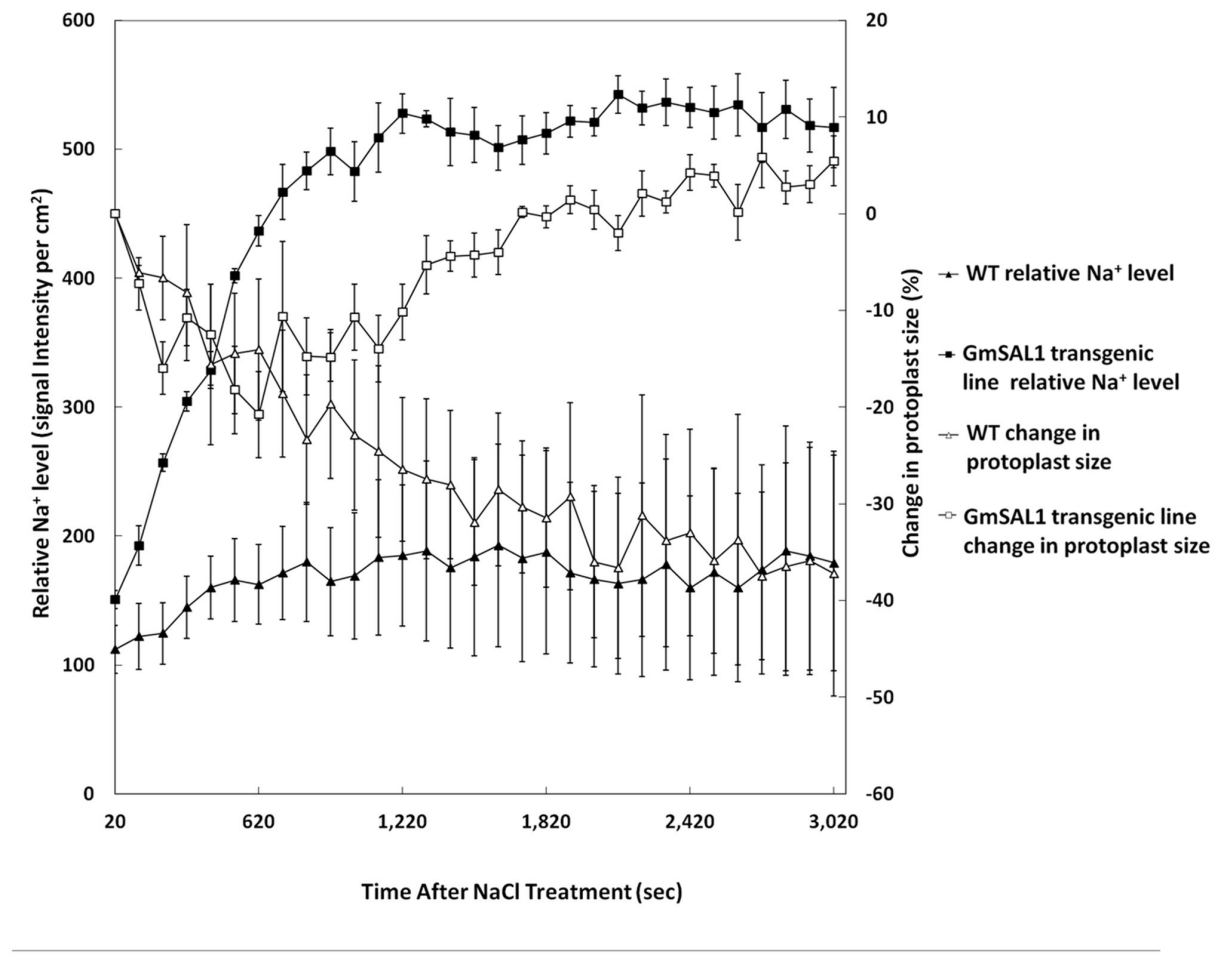
Changes in the protoplast size and vacuolar Na^+^ content under NaCl treatment in real time. *GmSAL1* transgenic BY-2 cells and the untransformed wild type cells (WT) were treated with 200mM NaCl (see Materials and Methods). Differential interference contrast (DIC) images and Sodium Green^TM^ fluorescent signals were collected by confocal microscopy (see Materials and Methods), over a period of 50min. Each data point represents the average value of 4-5 cells. Closed square: Na^+^ content of the *GmSAL1* transgenic line; closed triangle: Na^+^ content of WT; open square: protoplast size of the *GmSAL1* transgenic line; open triangle: protoplast size of the untransformed wild type (WT). Images were collated to produce the Video S1. The differences in signal intensity and cell size between WT and *GmSAL1* transgenic lines were subjected to the Student’s T-test. For Sodium Green signal comparison, p<0.01 from 20 sec after NaCl treatment till the end of the experiment (50 min and 20 sec). For cell size comparison, p<0.05 from 25 min and 20 sec after NaCl treatment till the end of the experiment.

To better visualize the changes of BY-2 cells under NaCl stress, we captured time-series images of a single cell after NaCl treatment. Two major differences were observed when comparing the wild type BY-2 cell to the *GmSAL1* transgenic cell. Firstly, when NaCl was added, the size of the protoplast in the wild type cell decreased (Figure 8; Video S1), probably due to the efflux of water from the cell. Under the same treatment, the *GmSAL1* transgenic cell exhibited an initial shrinkage in protoplast size followed by a recovery after about 15 min (Figure 8; Video S1). Secondly, while there was no significant elevation of Na^+^ compartmentalization into vacuoles over time in the wild type BY-2 cell under NaCl treatment, an obvious increase in vacuolar Na^+^ was observed in the *GmSAL1* transgenic cell under the same conditions. The vacuolar Na^+^ was maintained at a higher level than before the NaCl treatment when the transgenic cell gradually recovered from shrinkage (Figure 8; Video S1).

### Ectopic expression of *GmSAL1*did not enhance the tolerance of *A. thaliana* to NaCl and PEG stress

We also investigated the effect of ectopic expression of *GmSAL1* at whole plant level. Wild type (WT), transgenic empty vector (Empty vector), *GmSAL1* transgenic (GmSAL1-1 and GmSAL1-2) *A. thaliana* were treated with NaCl and near-isotonic PEG-6000. In contrast to the protective effects of GmSAL1 on BY-2 cells, ectopic expression of *GmSAL1* in *A. thaliana* did not confer obvious protection under salt stress and osmotic stress (Figure S3 in File S1).

## Discussion

It is common to classify enzymes that can act on I(1,4)P_2_ and I(1,3,4)P_3_ as inositol 1-phosphatase and those that can act on IP_3_ as inositol 5-phosphatase [5]. While GmSAL1 showed strong sequence homology to AtSAL1 and AtSAL2 which were reported to be inositol 1-phosphatases that have no activities toward IP_3_, GmSAL1 employed IP_3_ as the preferred substrate and is inactive toward I(1,4)P_2_ and I(1,3,4)P_3_ (Table 1).

There are two possible explanations for this observation. GmSAL1 may be an inositol 1-phosphatase like AtSAL1, but differs from AtSAL1 in substrate specificity. Another possibility is that GmSAL1 possesses inositol 5-phosphatase activities that act on the 5’-phosphate of IP_3_. Inositol polyphosphates without a 5’-phosphate such as I(1,4)P_2_ and I(1,3,4)P_3_ are therefore non-substrates. Detailed sequence analysis revealed that GmSAL1 exhibits a low degree of homology to the two consensus domains found in inositol 5-phosphatases (Figure S4 in File S1). In this research, we focus on the consequence of the IP_3_ hydrolytic activities exhibited by GmSAL1.

The K_m_ value of GmSAL1 toward IP_3_ was found to be about 10μM, which is similar to the K_m_ value of human inositol 5-phosphatase that also acts on IP_3_ [5]. This value is at least two folds higher than the cellular IP_3_ level that is needed to affect K^+^ transport [42].Therefore, GmSAL1 activity inside the cell may not be at maximum velocity under normal conditions. The IP_3_ level required to induce Ca^2+^ is at the μM level [42]. While mainly located in the cytosol, IP_3_ may be able to bind to receptors such as Ca^2+^ channels on the plasma membrane [30,43]. On the other hand, stress will increase the level of cytosolic IP_3_ [44–46]. For instance, NaCl treatment could increase the IP_3_ level up to 15 folds in *A. thaliana* [2]. GmSAL1 may therefore play a role in the fine adjustment of the cytosolic IP_3_ concentration under stress.

The expression of *GmSAL1* in its native host was responsive to NaCl (salinity stress) but not near-isotonic PEG treatment (osmotic stress) (Figure 2). This may be tied to its physiological roles. The ability of GmSAL1 to reduce the ABA-induced stomatal response (Figure 4), by lowering the IP_3_ level, is apparently not a protective mechanism against long-term osmotic stress. The expression of *GmSAL1* in transgenic BY-2 cells also showed no improvement in the tolerance toward PEG treatment (Figure S2 in File S1). On the other hand, GmSAL1 can help to combat salinity stress at the cellular level by enhancing the vacuolar compartmentalization of Na^+^ (Figure 6) and such an effect was not observed when IP_3_ was added. It is possible that under such experimental conditions, the protective function of GmSAL1 is brought forth by reducing the IP_3_ below a threshold level. 

 Using stomatal closure (Figure 4) and seed germination rate (Figure 5) as parameters, we showed that GmSAL1 can lower the plant’s sensitivity toward ABA treatments, presumably due to the reduction of IP_3_ signals. Such effects were also observed in inositol 5-phosphatases which use IP_3_ as their substrate [8,10]. 

The cytosolic IP_3_ offers protection against water loss *in planta* via inducing the closure of stomata by activating the tonoplast and cell membrane K^+^ channels that remove K^+^ from the vacuole and the cytosol and inactivating K^+^ channels that increase uptake [1]. These K^+^ channels are reported to be non-specific and can also transport Na^+^ [47,48]. Consistent with this observation, it was previously reported that the addition of NaCl could lead to stomatal opening, a phenomenon that could be reversed by ABA [49].

The classical model that IP_3_ is the direct signaling molecule inducing cytosolic Ca^2+^ influx in the guard cells [50] has been challenged by some recent findings. IP_6_ was found to be a much more potent signalling molecule controlling Ca^2+^ influx and the effect of IP_3_ on Ca^2+^ influx might be due to its conversion to IP_6_ [51]. However, GmSAL1 does not use IP_6_ as the substrate (Table 1) and hence the GmSAL1 effect on stomatal opening is via regulation of the cytosolic IP_3_ levels.

The level of cellular IP_3_increases under stress [44–46]. If the effect of IP_3_ on vacuolar cation channels also occurs in cells other than the guard cells, a higher level of IP_3_will decrease vacuolar Na^+^ compartmentalization. In contrast, the hydrolysis of IP_3_ will enhance the accumulation of vacuolar Na^+^ under NaCl treatments. It was indeed what we observed using the BY-2 cell model. GmSAL1 produced in the transgenic BY-2 cells hydrolyzed IP_3_ and hence increased Na^+^ compartmentalization in the vacuole (Figure 7), resulting in a higher survival rate for the transgenic cells under NaCl stress than for the wild type (Figure 6).

Compartmentalization of Na^+^ in the vacuole is an effective way to protect the plant cell against salinity stress [52–54]. On one hand, the toxic Na^+^ is removed from the cytosol. At the same time, the accumulation of Na^+^ in the vacuole sets up an osmotic gradient to enable the plant cell to uptake water from an environment with low osmotic potential [55,56]. This is supported by our observations that severe plasmolysis occurred in the untransformed wild type BY-2 cells upon NaCl treatment and the *GmSAL1* transgenic cells could accumulate Na^+^ in the vacuole more effectively and could therefore partially restore the protoplast size, presumably through increased water intake following ion compartmentalization in the vacuole (Figure 8; Video S1). The detailed mechanism of how GmSAL1 and cytosolic IP_3_ level regulate Na^+^ compartmentalization into vacuole is still unclear at this point.

Since ABA is the hormone which induces stomatal closure to protect the stressed plant from water loss [57] and GmSAL1 reduces ABA-induced stomatal response, it is not surprising that the protective effect of GmSAL1 on NaCl or PEG stress was not obvious at the whole plant level (Figure S4 in File S1).

In summary, we conclude that GmSAL1 is a novel soybean SAL1 homologue that hydrolyzes IP_3_ and plays differential roles at the whole plant level versus at the cellular level in response to salinity stress. 

## Supporting Information

File S1 **A combined file containing one supplemental table and four supplemental figures as follows: Table S1, Osmolarity of near-isotonic solutions; Figure S1, Validation of transgene expression; Figure S2, Ectopic expression of *GmSAL1* in BY-2 cells did not enhance their survival rates under PEG stress; Figure S3, Ectopic expression of *GmSAL1* in *A. thaliana* did not enhance their tolerance toward NaCl or PEG stress; Figure S4, Multiple alignments of GmSAL1 with inositol 5-phosphatases.**
(DOCX)

Video S1 **An MPEG video is provided to show the change in cell size and vacuolar Na^+^ in the *GmSAL1* transgenic and wild type BY-2 cell lines under 200 mM NaCl treatment.** The images were taken over a 50-min period.
(MPG)
