# Supplementary material for: GmSAL1 Hydrolyzes Inositol-1,4,5-Trisphosphate and Regulates Stomatal Closure in Detached Leaves and Ion Compartmentalization in Plant Cells
Source: PLoS One. 2013 Oct 22;8(10):e78181. doi: 10.1371/journal.pone.0078181 (PMC3805524; doi:10.1371/journal.pone.0078181)
Supplement: File S1 — A combined file containing one supplemental table and four supplemental figures as follows: Table S1, Osmolarity of near-isotonic solutions; Figure S1, Validation of transgene expression; Figure S2, Ectopic expression of GmSAL1 in BY-2 cells did not enhance their survival rates under PEG stress; Figure S3, Ectopic expression of GmSAL1 in A. thaliana did not enhance their tolerance toward NaCl or PEG stress; Figure S4, Multiple alignments of GmSAL1 with inositol 5-phosphatases. (DOCX) [file pone.0078181.s001.docx]

**Supporting Information**

**File S1.** A combined file containing one supplemental table and four supplemental figures as follows:

**Table S1.** Osmolarity of near-isotonic solutions.

**Figure S1.** Validation of transgene expression.

**Figure S2.** Ectopic expression of *GmSAL1* in BY-2 cells did not enhance their survival rates under PEG stress.

**Figure S3.** Ectopic expression of *GmSAL1* in *A. thaliana* did not enhance their tolerance toward NaCl or PEG stress.

**Figure S4.** Multiple alignments of GmSAL1 with inositol 5-phosphatases.

**Supplemental Video.** An MPEG video is provided to show the change in cell size and vacuolar Na^+^ in the *GmSAL1* transgenic and wild type BY-2 cell lines under 200 mM NaCl treatment. The images were taken over a 50-min period.

**Table S1. Osmolarity of near-isotonic solutions.**

| **Pairs of near-isotonic solutions in 1/2X Hoagland's solution** | | **Osmolarity (mOsm/L)** |
| --- | --- | --- |
| **NaCl concentration (mM)** | **PEG-6000 concentration (%) (w/v)** |  |
| 60 | 10 | 122+2.8 |
| 125 | 14 | 243+2.2 |
| 185 | 16.5 | 352+1.2 |
| **Pairs of near-isotonic solutions in MS medium for BY-2 cell suspension culture** | | **Osmolarity (mOsm/L)** |
| **NaCl concentration (mM)** | **PEG-6000 concentration (%) (w/v)** |  |
| 150 | 13.3 | 489+3.4 |
| **Pairs of near-isotonic solutions in MS medium for cultivation of *A. thaliana*** | | **Osmolarity (mOsm/L)** |
| **NaCl concentration (mM)** | **PEG-6000 concentration (%) (w/v)** |  |
| 100 | 11.1 | 381+1.2 |
| 150 | 13.5 | 464+1.6 |

The osmolarity is the average value of three measurements of NaCl solution and PEG solution + standard error of the six measurements. The osmolarity was measured by Advanced^TM^ Micro Osmometer (Model 3300; Advanced Instruments, Inc., Norwood, MA, USA).

**
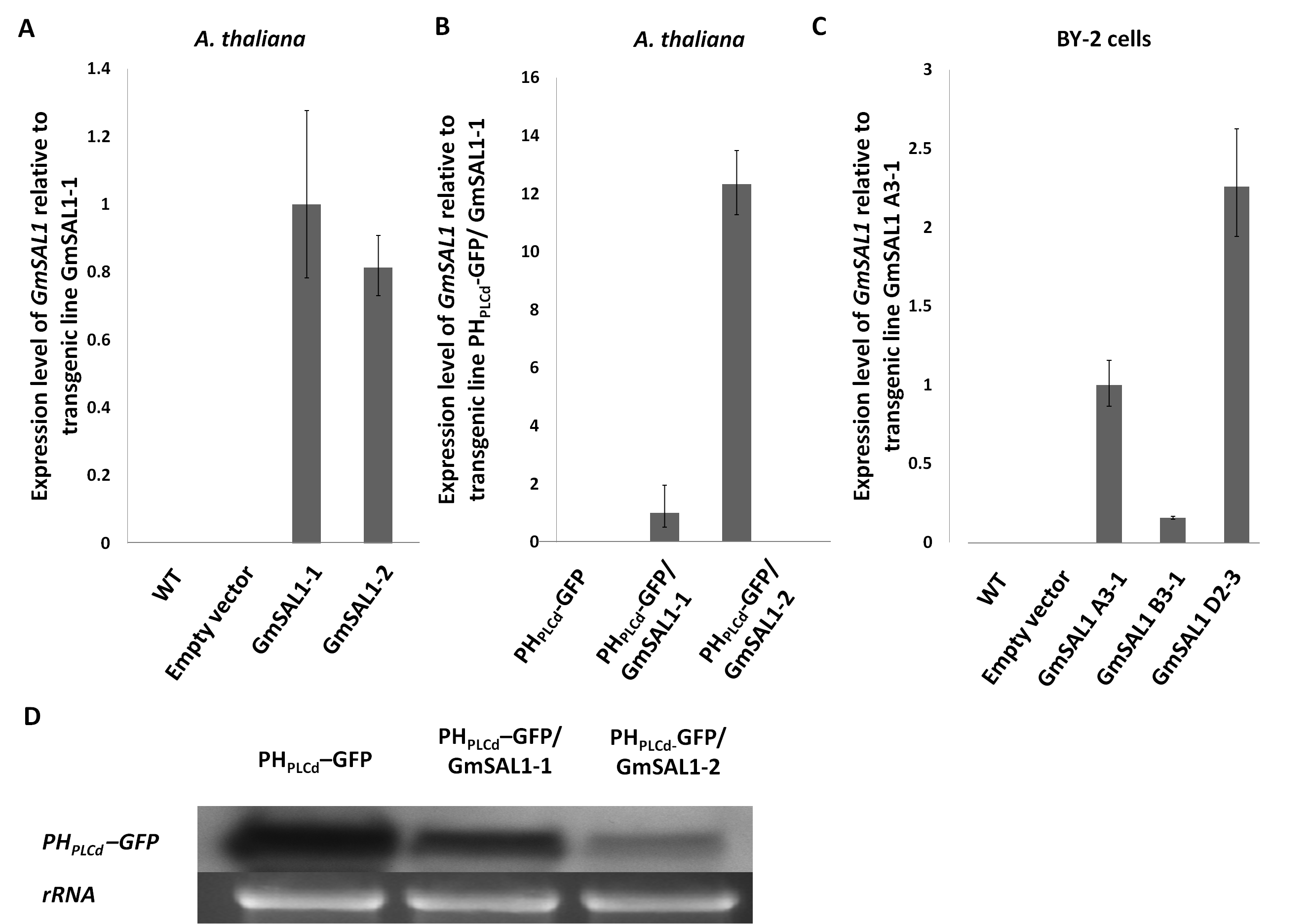
**

**Figure S1. Validation of transgene expression.**

The expression of *GmSAL1* in transgenic *A. thaliana* and BY-2 cells was verified by real-time PCR (A-C) or northern blot analysis (D). Samples are from: **A:** Two-week-old untransformed control (WT), empty-vector transgenic control (Empty vector), and two independent transgenic *A. thaliana* lines (*GmSAL1-1* and *GmSAL1-2*) grown on MS agar plates; **B:** Two-week-old *PH_PLCd_–GFP* and two independent double-transformed (*PH_PLCd_–GFP*/*GmSAL1-1* and *PH_PLCd_–GFP*/*GmSAL1-2*) *A. thaliana* lines grown on MS agar plates; **C:** Four-day-old untransformed control (WT), empty-vector transgenic control (Empty vector), and three independent *GmSAL1* transgenic BY-2 cell lines (A3-1, B3-1, D2-3) grown on MS medium. All real-time PCR experiments were triplicated. Error bar: standard error. **D:** Rosette leaves of four-week-old *A. thaliana* grown on soil.

**
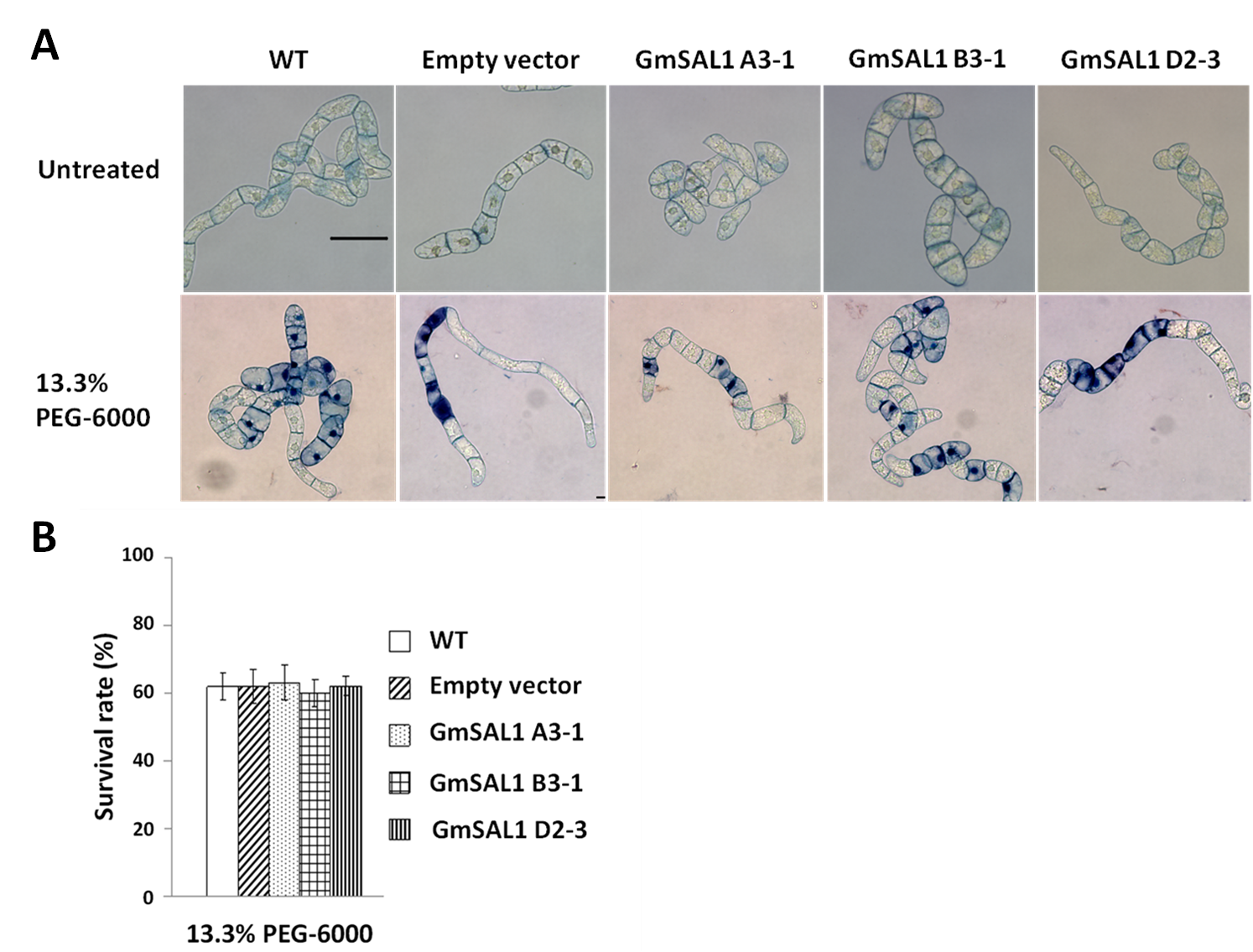
**

**Figure S2. Ectopic expression of *GmSAL1* in BY-2 cells did not enhance their survival rates under PEG stress.**

Four-day-old BY-2 cells grown in MS medium were used, including cells of untransformed wild type (WT), empty-vector transgenic control (Empty vector), and independent *GmSAL1* transgenic lines (A3-1, B3-1, D2-3). **A:** Cell death is visualized by Trypan blue staining. The cells were either untreated, or treated with PEG-6000 (13.3%) (near-isotonic to 150mM NaCl) in MS for 24 h with shaking. They were then washed with fresh MS medium and stained with 0.4μg/μl Trypan blue for 15 min before microscopic analyses. Scale bar = 100μm. **B:** Statistical analyses of the effect of PEG stress on survival rates. A total of 92-329 cells were counted from 7-12 fields. Error bar: standard error.


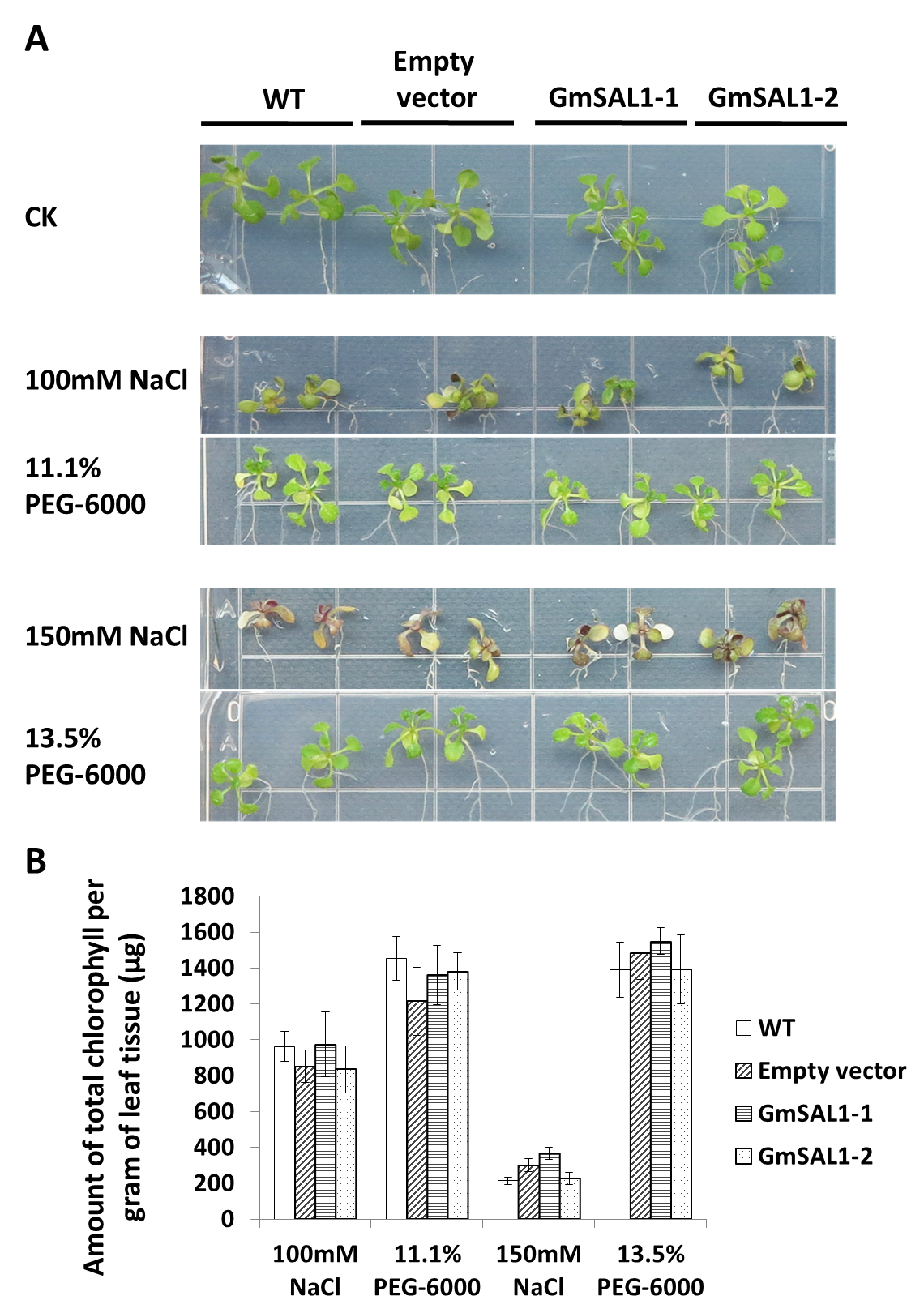


**Figure S3. Ectopic expression of *GmSAL1* in *A. thaliana* did not enhance their tolerance toward NaCl or PEG stress.**

The stress treatments were performed as described in previous reports [34,35] with slight modifications. Ten-day-old *A. thaliana* seedlings grown on MS agar at 22°C (16 h light-8 h dark cycle) were transferred to MS agar without supplement (CK) or MS agar supplemented with 100 mM NaCl, 11.1% (w/v) PEG-6000, 150 mM NaCl, or 13.5% (w/v) PEG-6000 (100 mM NaCl MS broth is near-isotonic to 11.1% (w/v) PEG-6000 MS broth; 150mM NaCl MS broth is near-isotonic to 13.5% (w/v) PEG-6000 MS broth). GmSAL1-1 and GmSAL1-2 are two independent transgenic lines. **A:** Images were taken 10 days after treatment. **B:** Statistical analyses of the amount of total chlorophyll of NaCl or PEG treated *A. thaliana*. The determination of total chlorophyll in *A. thaliana* was performed as described previously [34]. Leaf tissue of less than 0.02 g was immersed in 0.8 ml N, N-dimethylformamide (DMF) followed by incubation at 4°C overnight [36]. The absorbances at 603, 647 and 664 nm were recorded. The amount of total chlorophyll was calculated using a formula published previously [37]. A total of 6 seedlings were harvested from 3 plates. Error bar: standard error.

**
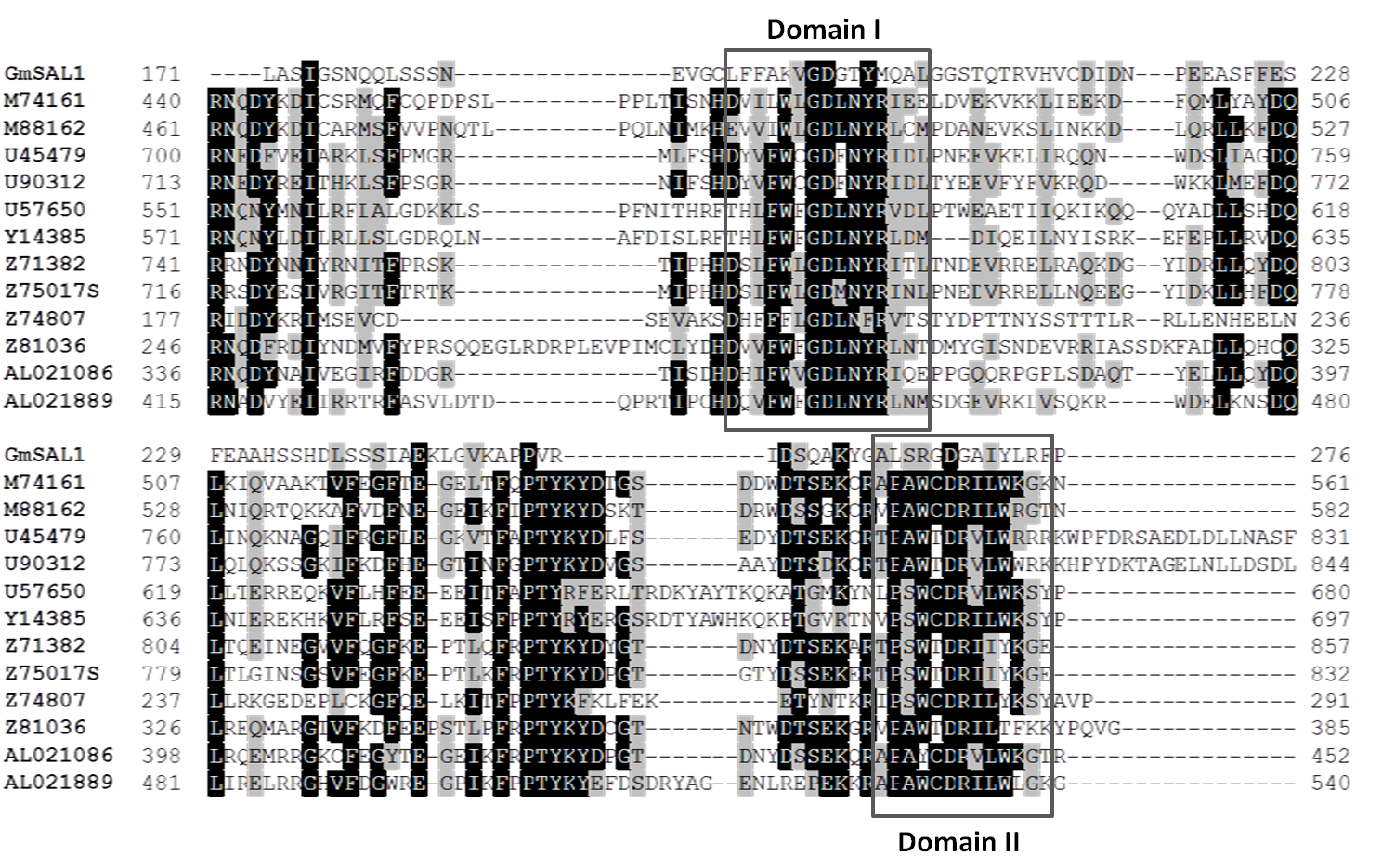
**

**Figure S4. Multiple alignments of GmSAL1 with inositol 5-phosphatases.**

The amino acid sequence of GmSAL1 was aligned with inositol 5-phosphatases from various organisms, including human (M74161, M88162, U57650, Y14385), *Rattus norvegicus* (U45479, U90312), *Saccharomyces cerevisiae* (Z7138, Z7501, Z7480), *Caenorhabditis elegans* (Z81036), *Drosophila melanogaster* (AL0210), and *Arabidopsis thaliana* (AL021889). The alignment was performed using the built-in ClustalW in the program BioEdit (ver.7.0.5.3). Identical amino acid residues were shaded black and similar amino acid residues were shaded grey. The alignment revealed that GmSAL1 exhibits a low degree of homology to the two consensus domains (Domain I and Domain II) found in inositol 5-phosphatases [4].
